# Supplementary material for: Centrosome, the Newly Identified Passenger through Tunneling Nanotubes, Increases Binucleation and Proliferation Marker in Receiving Cells
Source: Int J Mol Sci. 2021 Sep 7;22(18):9680. doi: 10.3390/ijms22189680 (PMC8467045; doi:10.3390/ijms22189680)
Supplement: Supplementary file 1 [file ijms-22-09680-s001.zip › Figures supp TNT and centrosome VDEF 26 August.pdf]

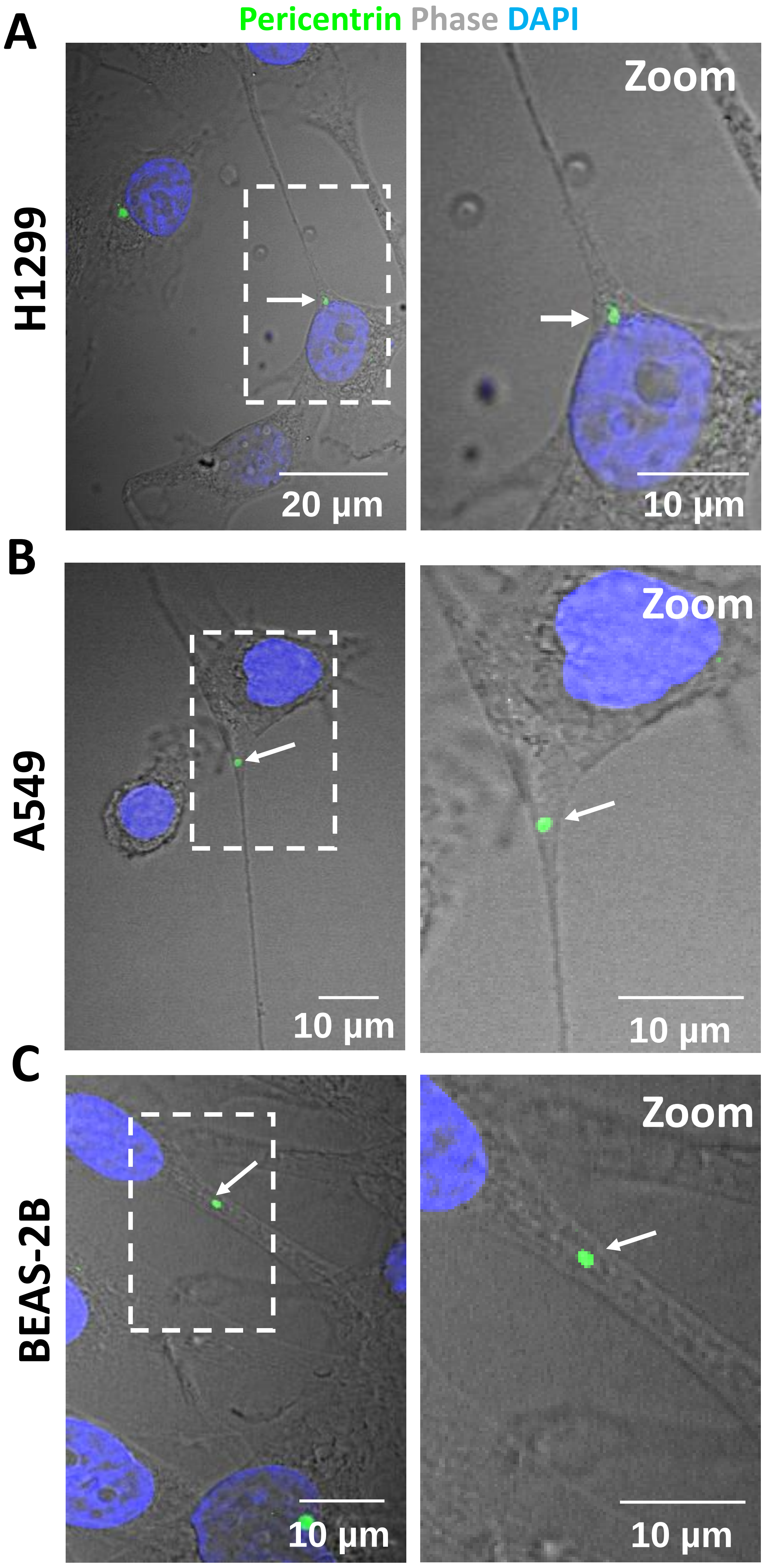

Figure 1 supplementary: The centrosome polarizes towards TNT-1 protrusion sites. Representative images of centrosome orientation towards TNTs-1 formation site in H1299 (**A**), A549 (**B**) and BEAS-2B (**C**), three tumorigenic epithelial cell lines. Arrows indicate centrosome position. Boxed regions used for the zoom.

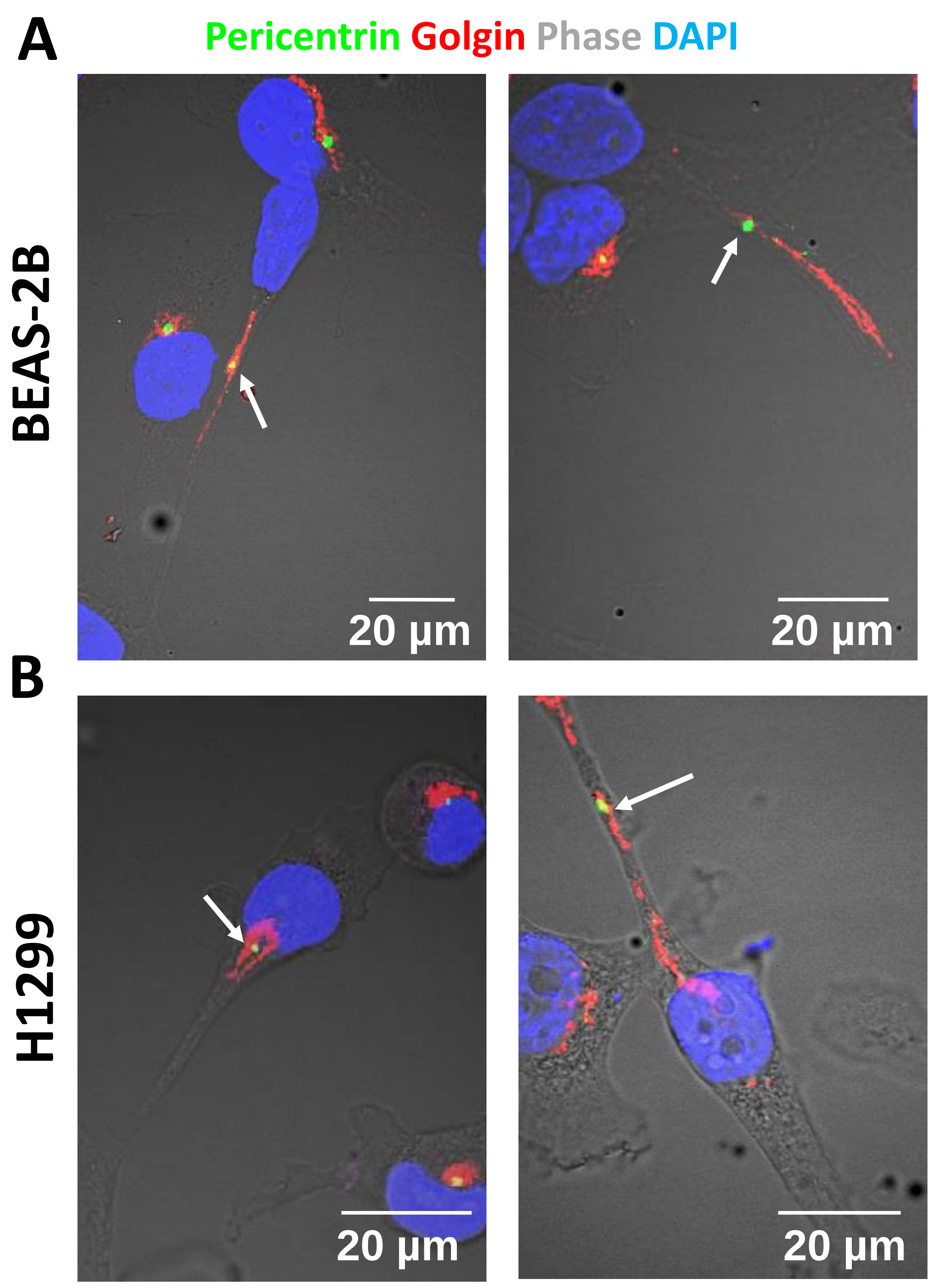

Figure 2 Supplementary: The pericentrosomal localization of the Golgi towards the site of TNT-1 formation. Two representative images of pericentrosomal Golgi orientation towards TNTs-1 formation site in BEAS-2B (**A**) and H1299 (**B**), two tumorigenic epithelial cell lines. Arrows indicate centrosome position.

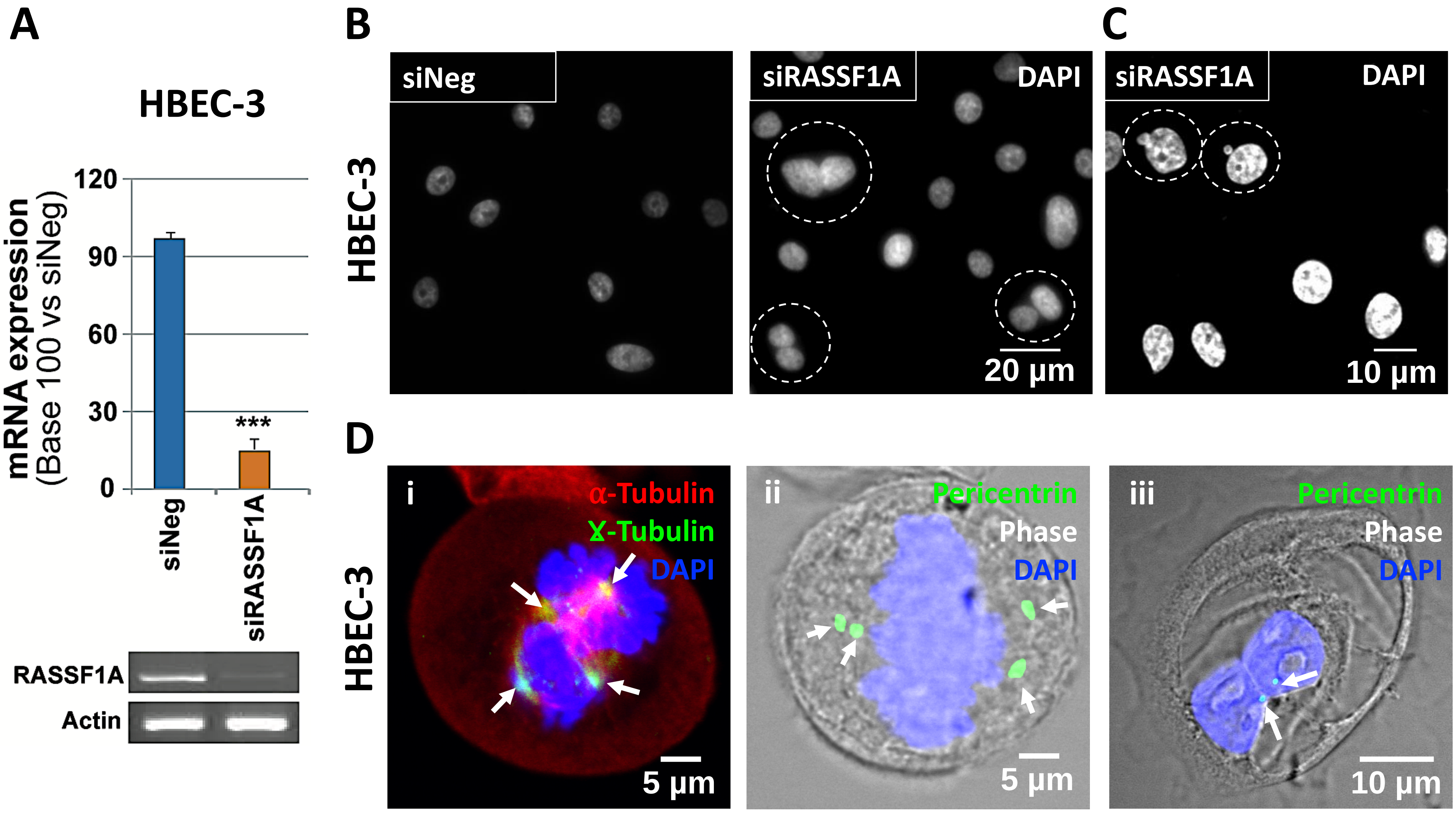

Figure 3 supplementary: In results of centrosome abnormalities, RASSF1A depletion reduces the fidelity of DNA segregation. A) Evaluation of the efficiency of RASSF1A RNAi by RT-qPCR**.** Representative images of nuclear atypia such as **B**) bi-nucleation (indicated by dashed circles) and **C**) micronuclei, as an indication of **D**) extra centrosome (i, ii) and mitotic spindle defects (iii). HBEC-3 cells transfected with either siNeg or siRASSF1A. Experiments were performed 72 hours after transfection. Arrows indicate centrosome position.

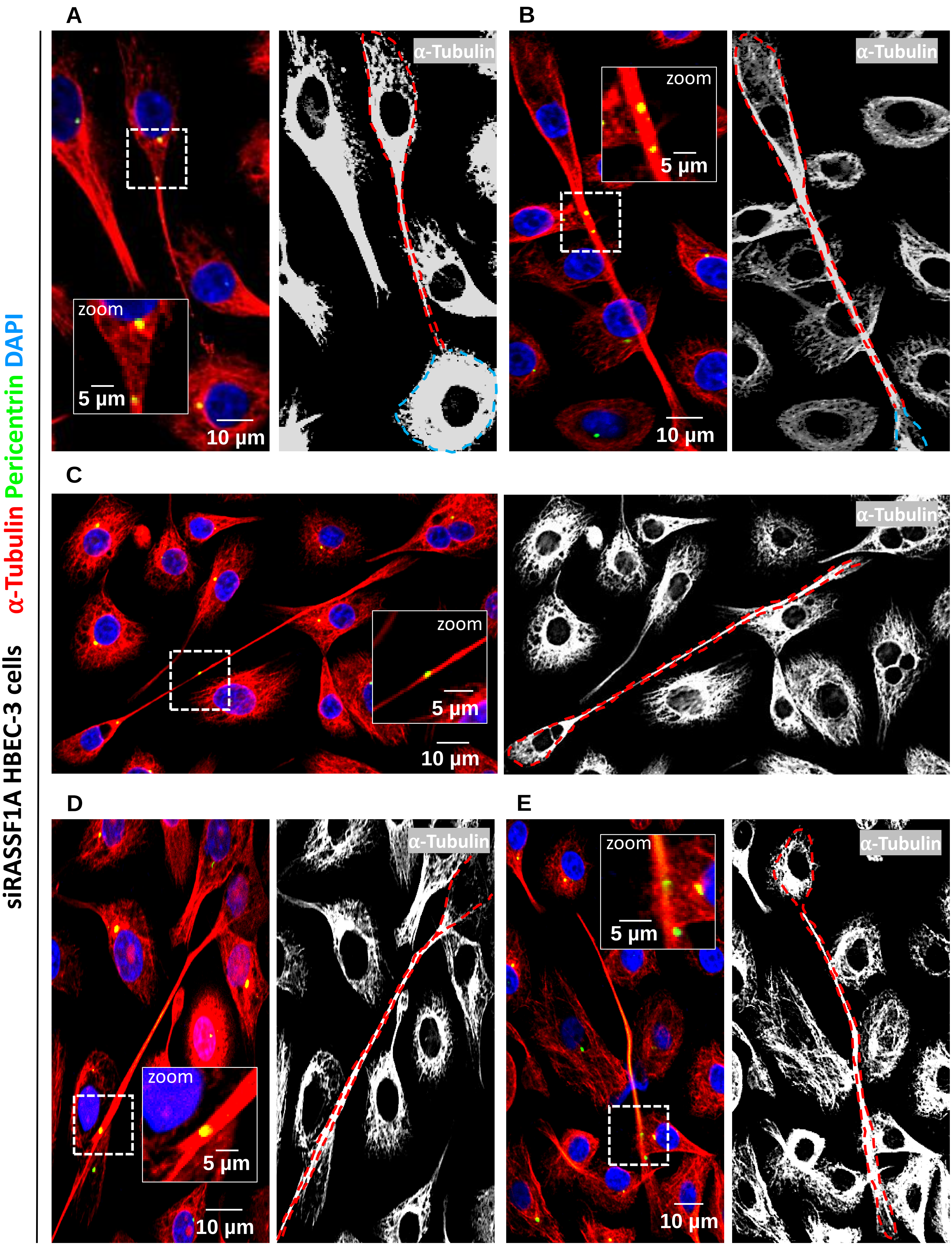

Figure 4 supplementary: Centrosome localization within TNTs-1. Representative images (**A-E**) of siRASSF1A-depleted HBEC-3 cells, showing centrosome localization within TNT-1 in HBEC-3 cell lines**. Dashed red lines represent the perimeter of cells with TNT-1 and boxed regions used for the zoom.**

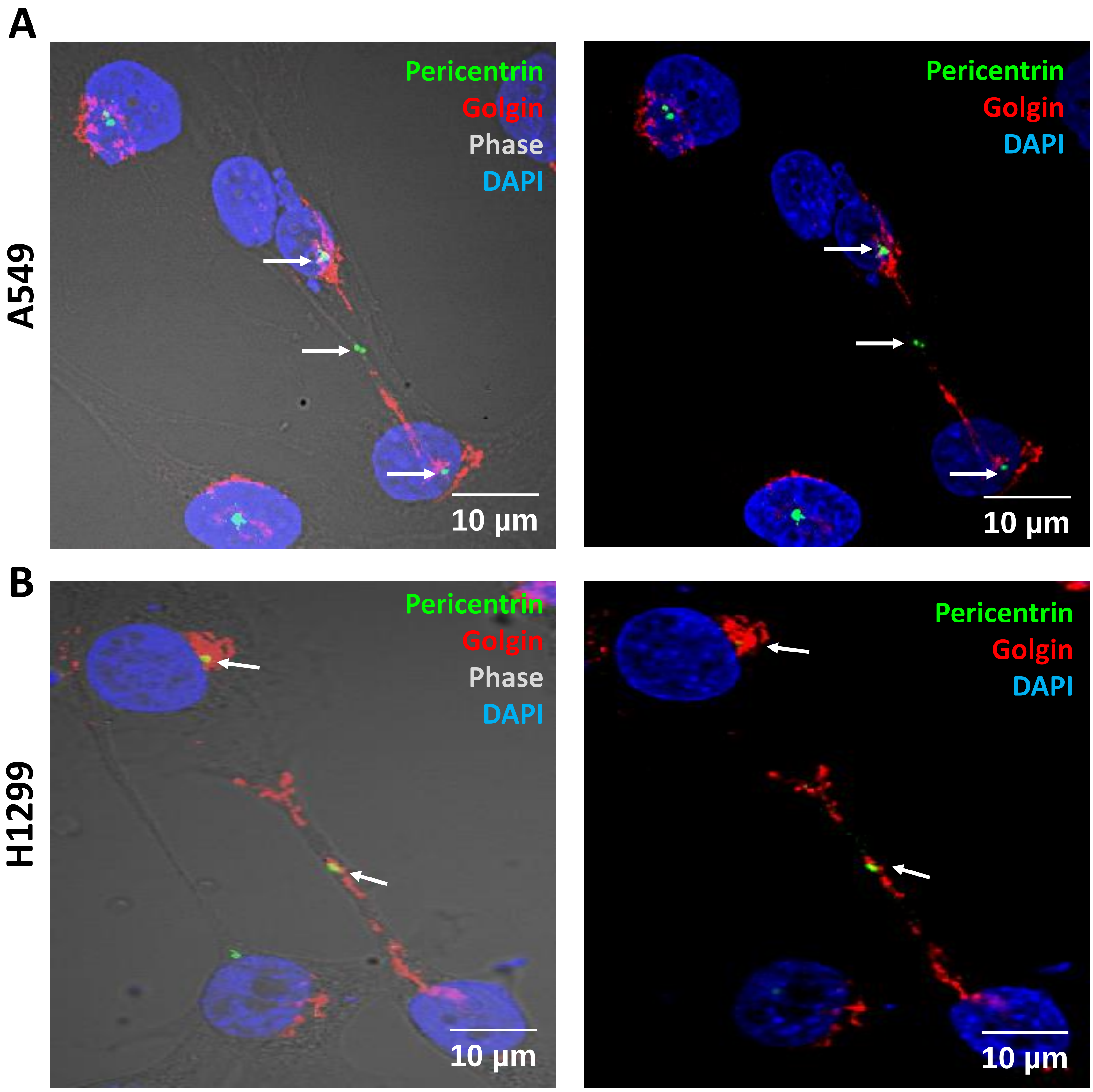

Figure 5 supplementary: Centrosome and Golgi localization within TNTs-1. Representative images showing centrosome displacement through TNT-1 in lung epithelial A549 (**A**) and H1299 (**B**) tumorigenic cell lines with hypermethylated *RASSF1* gene**.** Arrows indicate centrosome position.

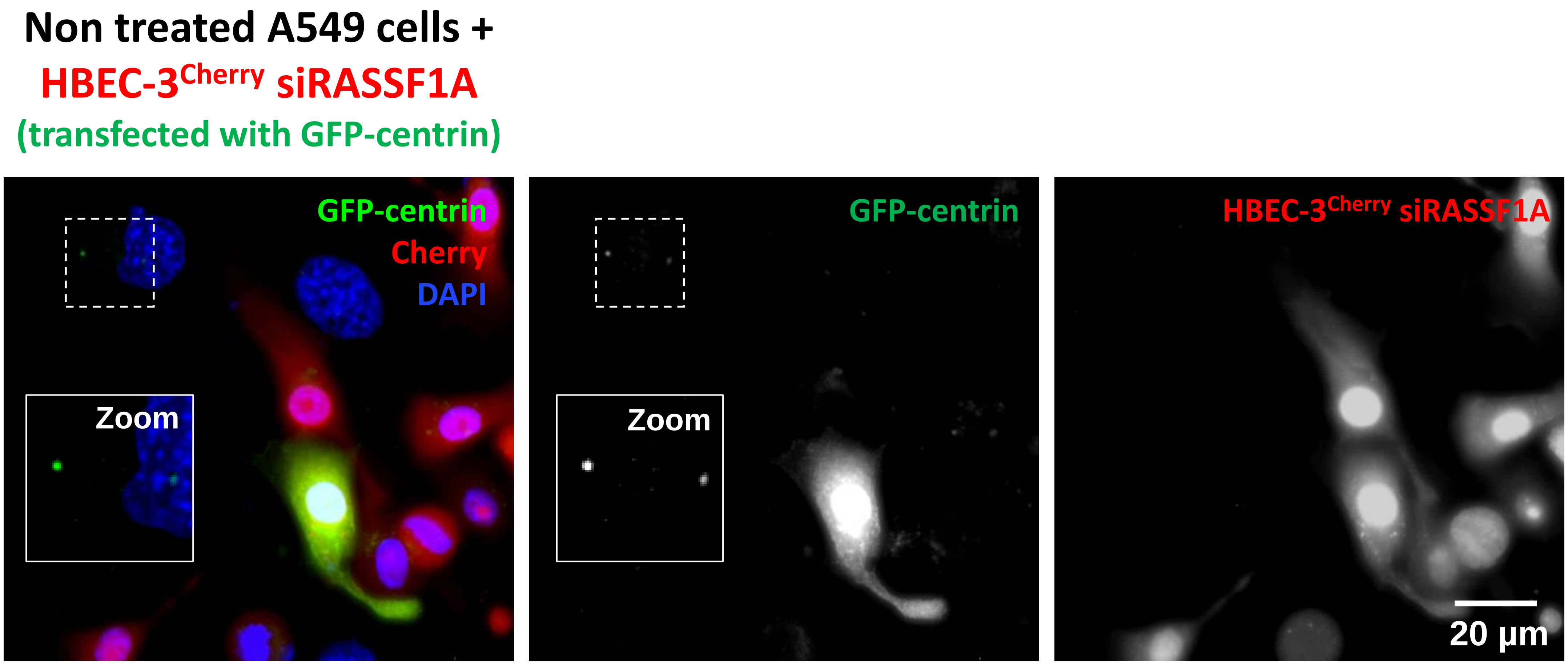

Figure 6 supplementary: Representative images showing displacement of centrosome (signal of GFP-centrin) from donor RASSF1A-depleted GFP-centrin transfected HBEC-3 cherry cells to acceptor non-treated A549 cells. Boxed regions used for the zoom.
