# Supplementary figures and images for: Centrosome, the Newly Identified Passenger through Tunneling Nanotubes, Increases Binucleation and Proliferation Marker in Receiving Cells
Source: Int J Mol Sci. 2021 Sep 7;22(18):9680. doi: 10.3390/ijms22189680 (PMC8467045; doi:10.3390/ijms22189680)

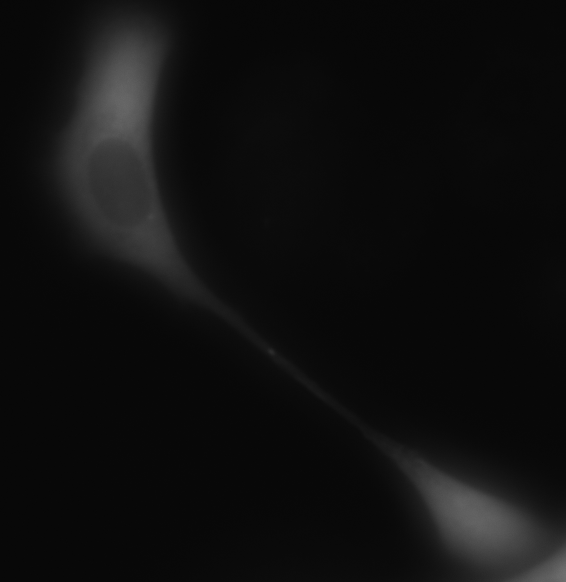

Supplement: Supplementary file 1 [file ijms-22-09680-s001.zip › Movie 1.tif]
